# Inflammatory Markers in Women with Polycystic Ovary Syndrome

**DOI:** 10.1155/2020/4092470

**Published:** 2020-03-04

**Authors:** E. Rudnicka, M. Kunicki, K. Suchta, P. Machura, M. Grymowicz, R. Smolarczyk

**Affiliations:** ^1^Medical University of Warsaw Poland, Clinic for Gynecological Endocrinology, Poland; ^2^Invicta Infertility Center, Warsaw, Poland; ^3^Department of Gynecological Endocrinology, Karowa Hospital, Warsaw, Poland

## Abstract

Several studies have reported the association between polycystic ovary syndrome (PCOS) and low-grade chronic inflammation to be of uncertain cause: obesity, insulin resistance, or PCOS itself. The aim of the study was to investigate the WBC (white blood cell) count and CRP (C-reactive protein) concentration in women with PCOS and to determine the factors that affect their concentration. The study included 200 women aged 18-40 with PCOS and 105 healthy women as the control group, recruited in the Department of Gynaecological Endocrinology of Medical University in Warsaw from 2016 to 2018. Each patient underwent clinical, biochemical, and ultrasonographic assessments. WBC and CRP were significantly higher in the PCOS group (*Z* = −2,353, *p* = 0,019 and *Z* = −2,453, *p* = 0,014). WBC positively correlated with serum insulin at 0, 60, and 120 min during the oral glucose tolerance test (INS0: *r* = 0,221, *p* = 0,001; INS1: *r* = 0,194, *p* = 0,003; INS2: *r* = 0,022, *p* = 0,001), testosterone (*r* = 0,130, *p* = 0,046), androstenedione (*r* = 0,212, *p* = 0,001), and DHEAS (*r* = 0,178, *p* = 0,006) and negatively correlated with progesterone (*r* = −0,204, *p* = 0,002), estradiol (*r* = −0,140, *p* = 0,032), and SHBG (*r* = −0,308, *p* < 0,001). CRP positively correlated with insulin concentration in 0, 60, and 120 min during the oral glucose tolerance test (INS0: *r* = 0,343, *p* < 0,001; INS1: *r* = 0,276, *p* = 0,001; INS2: *r* = 0,320, *p* < 001) and negatively correlated with progesterone (*r* = −0,194, *p* = 0,030) and SHBG (-0,244, *p* = 0,005). We also estimated positive correlation between BMI and serum CRP and WBC concentration. Multiple linear regression analysis showed that CRP values are positively associated with BMI (beta = 0,374, *p* < 0,001) and insulin level (INS1) (beta = 0,282, *p* = 0,004); and WBC results are negatively associated with SHGB (beta = −0,284, *p* < 0,001) but positively associated with testosterone (beta = 0,163, *p* = 0,024) and BMI (beta = 0,157, *p* = 0,047). PCOS is associated with increased WBC and CRP concentrations. The main predicting factors of increased CRP are BMI and insulin resistance, but there is also a relationship between WBC count in PCOS and androgen concentration itself so that inflammation may be mediated not only through adiposity but also through increased androgen concentration.

## 1. Introduction

Polycystic ovary syndrome (PCOS) is the most common endocrine disorder, which affects 10-15% of women in their reproductive age [1]. It is characterized by oligo-/anovulation, clinical or biochemical hyperandrogenism, and polycystic ovaries identified by ultrasound examination [1, 2]. PCOS has significant clinical implications, including reproductive and metabolic disorders, such as hyperinsulinemia, glucose intolerance, abnormal blood lipid levels, and obesity [3, 4]. Recently, there is a rise of numerous inflammatory markers observed in women with PCOS. In these patients, elevation in white blood cell (WBC) count, C-reactive protein (CRP), and some cytokine concentrations, including interleukin 6 (IL-6), interleukin 18 (IL-18), and tumor necrosis factor-*α* (TNF-*α*), is found [5–7]. Chronic low-grade inflammation has been implicated as a risk factor of endothelial dysfunction, atherosclerosis, and coronary heart disease and is linked to insulin resistance (IR) and abdominal obesity [8, 9]. Several studies have reported on the association between PCOS and low-grade chronic inflammation, but there is still uncertainty about the causal relationship: obesity, insulin resistance, or the polycystic ovarian syndrome itself [10–12]. It has been postulated that there are also proinflammatory effects of hyperandrogenemia and that the inflammatory state in PCOS women is greater than that resulting from IR and obesity alone [13, 14].

The aim of the present study was to investigate the white blood cell count and CRP concentrations in women with PCOS, to compare them to age- and BMI-matched healthy controls, and to determine the factors that affect WBC and CRP concentrations in this group.

## 2. Materials and Methods

The study included 200 women with polycystic ovary syndrome in the 18-40 age range and 105 healthy control women, recruited in the Department of Gynaecological Endocrinology of Medical University in Warsaw in the years 2016-2018. We obtained approval for our study from the Ethics Committee of Medical University of Warsaw in Poland. Informed consent was obtained from all participants. The diagnosis of PCOS was based on Rotterdam consensus and association of at least two of following criteria: (1) oligo- or anovulation, (2) clinical or/and biochemical hyperandrogenism, and (3) polycystic morphology of ovary in ultrasound examination [1]. PCOS subjects and controls were matched according to age and body mass index (BMI). The body mass index was calculated based on measurement of height and weight by a single observer and not on self-reporting using the following formula: weight (kg)/height (m^2^). The patients presented with hirsutism were assessed according to Ferriman-Gallwey score (>7) [15]. Control subjects were recruited from healthy women admitted to our outpatient clinic for periodic medical examinations. The control subjects meet the following criteria: regular menstruation (25-35 days) and without any sings of hyperandrogenism. Exclusion criteria were as follows: pregnancy, breastfeeding, other hyperandrogenemic conditions (Cushing syndrome, late-onset congenital adrenal hyperplasia, and androgen-secreting tumors), known diabetes mellitus, genetic and acquired hematological diseases, thyroid dysfunction, fever (defined as body temperature higher than 38°C), and viral and bacterial illnesses. Oral contraceptives or other drugs (for example, metformin) that could interfere with the hormonal and metabolic studies were discontinued for at least 3 months before the study. For excluding Cushing's syndrome, a 1 mg dexamethasone suppression test was performed: the patient got 1 mg dexamethasone at 11 PM and the cortisol concentration was measured at 8 PM the day after. Serum cortisol at 8 AM (<1,8 *μ*g/l) excluded Cushing's syndrome. Increased 17-OHP was defined as serum concentration of 17‐OHP > 2 ng/ml. When serum 17-OHP was between 2 and 10 mg/l, an adrenocorticotropic hormone (ACTH) stimulating test was performed for excluding nonclassic adrenal hyperplasia (21-hydroxylase deficiency) [1, 16].

Each patient underwent clinical, biochemical, and ultrasonographic assessments. Blood samples were collected between 8 and 10 AM after an overnight fast. Basal serum levels of follicle-stimulating hormone (FSH), luteinizing hormone (LH), estradiol (E2), prolactin (PRL), thyrotropin (TSH), free thyroxine (fT4), total testosterone (tT), sex hormone binding globulin (SHBG), androstenedione (A), dehydroepiandrosterone (DHEAS), and 17 alpha-hydroxyprogesterone (17-OHP) were obtained during early follicular phase of the menstrual cycle (between 3-6 day) and progesterone between days 22 and 24 counting from the first day of menstruation. Standard 75 g oral glucose tolerance test (OGTT) and insulin level measurement were performed in all patients to evaluate insulin resistance. A lipid profile (total cholesterol, high-density lipoprotein (HDL) cholesterol, low-density lipoprotein (LDL) cholesterol, triglyceride (TG), and full blood count, including differential white cell count and C-reactive protein (CRP)) was assessed for each subject. Pelvic ultrasound was performed using transvaginal ultrasound transducer Aloka Alpha7, and ovarian morphologic features were assessed. The ovaries were scanned in both the anterior-posterior and transverse cross-section dimensions, from the inner to the outer margins. Ovarian volume and outlines were calculated by the machine software. Polycystic ovaries were defined by the presence of 12 or more follicles 2-9 mm in diameter or/and increased ovarian volume > 10 mm^3^.

### 2.1. Laboratory

Our laboratory normal reference ranges during the follicular phase were as follows: FSH: 3,03-8,08 mIU/ml; LH: 1,8-11,78 mIU/ml; estradiol: 21-251 pg/ml; prolactin: 5-35 ng/ml; TSH: 0,35-4,94 *μ*mol/ml; fT4: 9,01-19,05 pmol/l; androstenedione: 0,3-3,5 ng/ml; DHEAS: 2,68-9,23 *μ*mol/l; testosterone: 0,1-0,56 ng/ml; SHBG: 19,84-155,2 nmol/l; and 17-OHP: 0,3-1,0 ng/ml. Anovulation was defined as serum progesterone level < 3 ng/ml. Serum FSH, LH, TSH, fT4, E2, PRL, T, and SHBG were measured using an enzyme-linked fluorescent assay (ELFA) (VIDAS, bioMérieux). 17-OHP levels were measured using an enzyme-linked immunosorbent assay (ELISA) (EUROIMMUN AG Analyzer I). The serum concentration of androstenedione was tested using chemiluminescent immunoassay technique (IMMULITE 2000XP, Siemens Healthineers). Serum insulin and cortisol were measured using a chemiluminescent microparticle immunoassay (CMIA) (Architect i2000SR, Abbott Diagnostics). Serum glucose, total cholesterol, triglycerides, high-density lipoprotein (HDL) cholesterol, and low-density lipoprotein (LDL) cholesterol were analysed using an enzymatic colorimetric method (Konelab Prime 301 by Thermo Scientific).

### 2.2. Statistical Analysis

The statistical analysis was performed using IBM SPSS Software, version 20. The Shapiro-Wilk test was performed to test for normality. Differences between PCOS patients and the control group were compared with the nonparametric Mann-Whitney *U* test. Spearman's correlation coefficients were used to test the correlation between WBC, CRP, and hormone profile and glucose and insulin concentrations. To establish the correlation between BMI and WBC and CRP, Pearson's correlation was applied. In order to determine the influence of several variables on the CRP and WBC results, stepwise linear regression was performed. *p* value < 0,05 was determined as statistically significant.

## 3. Results

Anthropometric characteristics and the main hormonal and metabolic profile of PCOS patients and the control group are presented in Table 1. There were no statistically significant differences in age and BMI between the two groups.

White blood count (WBC) was significantly higher in the PCOS group (*N* = 200), compared with the healthy controls (*Z* = −2,353, *p* = 0,019). We also observed statistically significant differences in CRP concentrations between the PCOS group and controls (*Z* = −2,453, *p* = 0,014).

Women with PCOS presented with significantly higher serum levels of LH (*Z* = −5,145, *p* < 0, 01), testosterone (*Z* = −6, 4441, *p* < 0,001), androstenedione (*Z* = −6,983, *p* < 0, 0010), DHEAS (*Z* = −6,631, *p* < 0,001), and 17-OHP (*Z* = −6,939, *p* < 0,001) and lower FSH (*Z* = −2,253, *p* = 0,024), estradiol (*Z* = −2,756, *p* = 0,006), SHBG (*Z* = −3,592, *p* < 0,001), and progesterone (*Z* = −4,225, *p* < 0,001), respectively.

Regarding metabolic parameters, patients with PCOS had significantly higher serum concentrations of LDL cholesterol (*Z* = −5,158, *p* < 0,001), glucose, and insulin 1 h and 2 h during the oral glucose tolerance test (glucose 1 h: *Z* = −2,395, *p* = 0,017; glucose 2 h: *Z* = −2,174, *p* = 0,030; insulin 1 h: *Z* = −2,882, *p* = 0,004; insulin 2 h: *Z* = −3,513, *p* < 0,001) and significantly lower concentrations of HDL cholesterol (*Z* = −5,268, *p* < 0,001).

**Figure 1:**
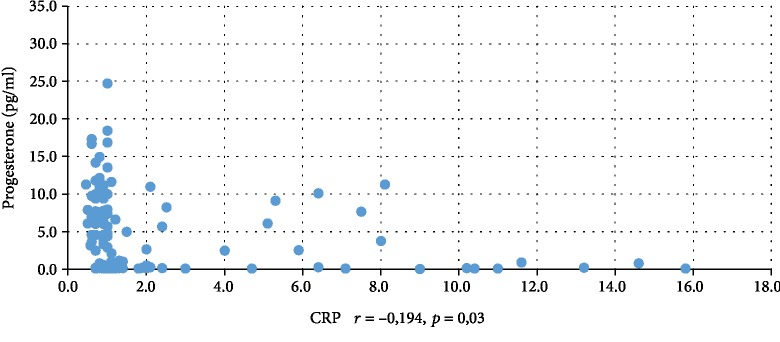
Correlation between progesterone and CRP.

**Figure 2:**
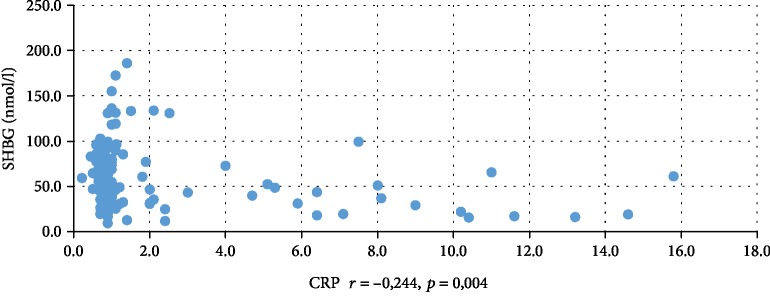
Correlation between SHBG and CRP.

**Figure 3:**
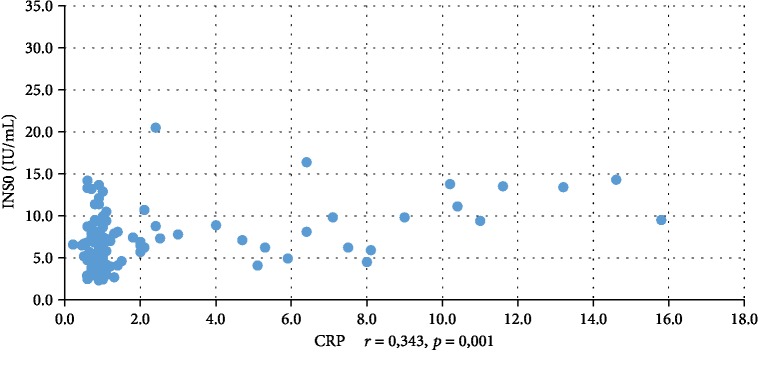
Correlation between INS0 and CRP.

**Figure 4:**
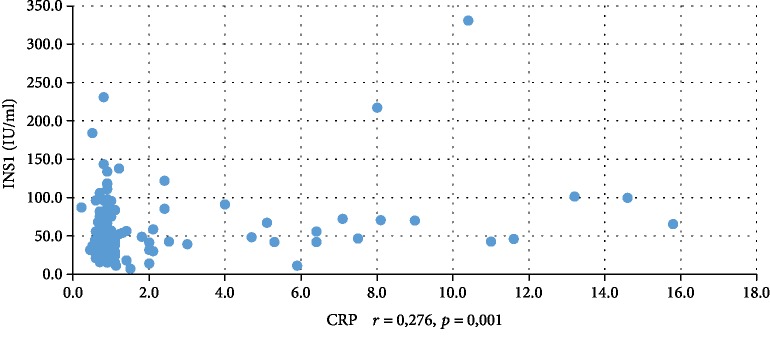
Correlation between INS1 and CRP.

**Figure 5:**
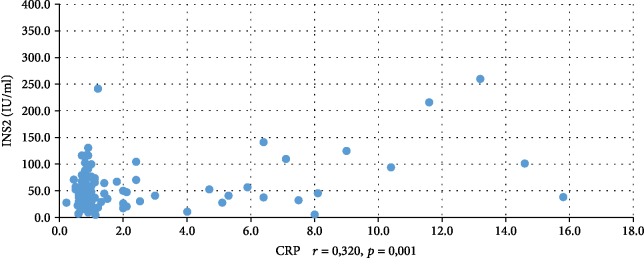
Correlation between INS2 and CRP.

**Figure 6:**
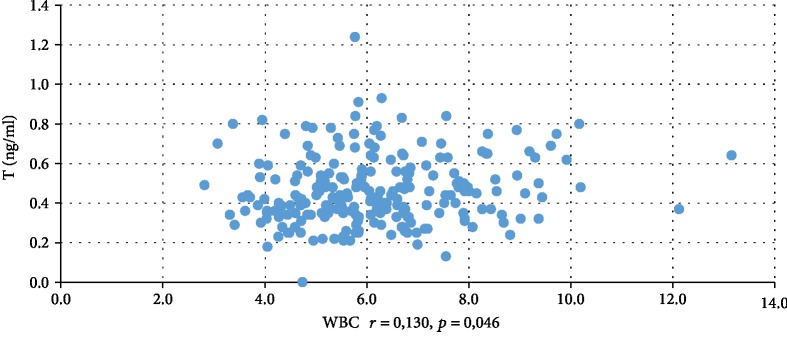
Correlation between testosterone and WBC.

**Figure 7:**
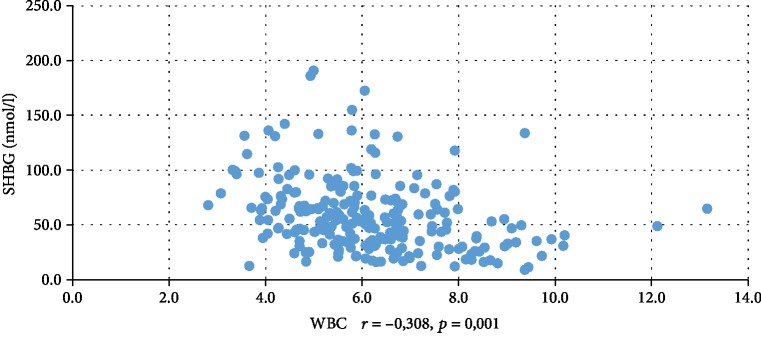
Correlation between SHBG and WBC.

**Figure 8:**
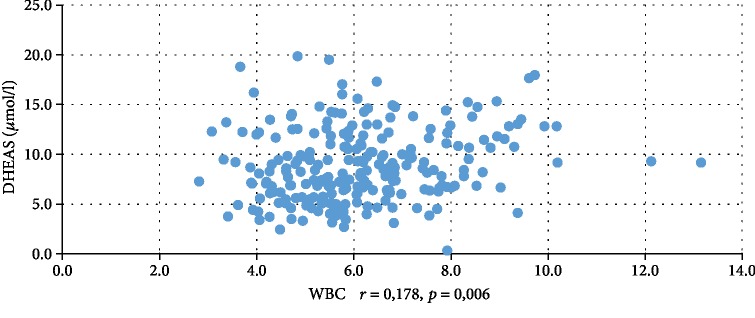
Correlation between DHEAS and WBC.

**Figure 9:**
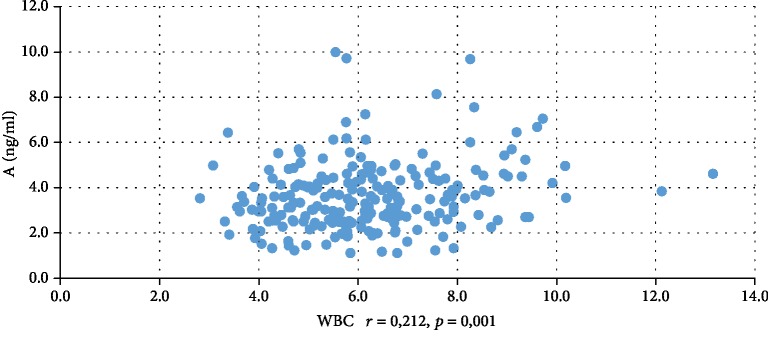
Correlation between androstenedione and WBC.

**Figure 10:**
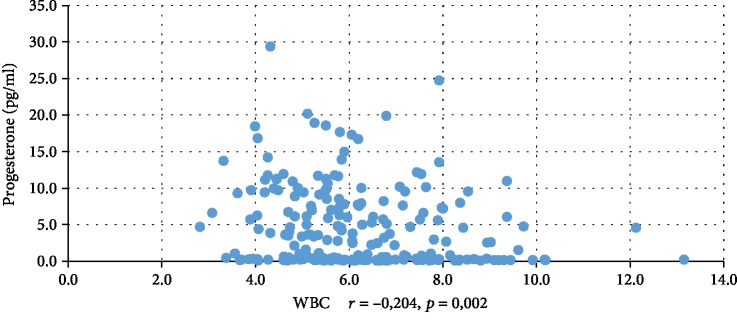
Correlation between progesterone and WBC.

**Figure 11:**
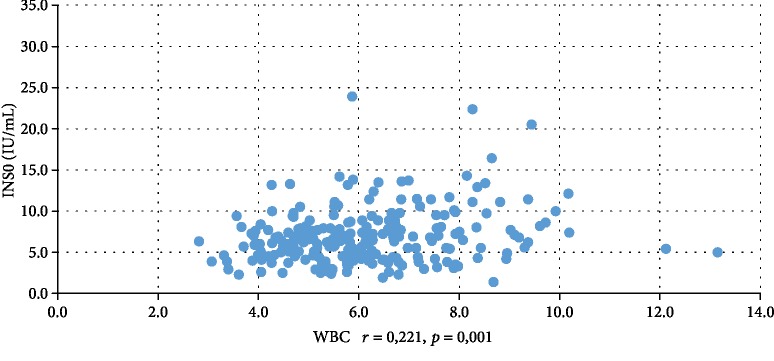
Correlation between INS0 and WBC.

**Figure 12:**
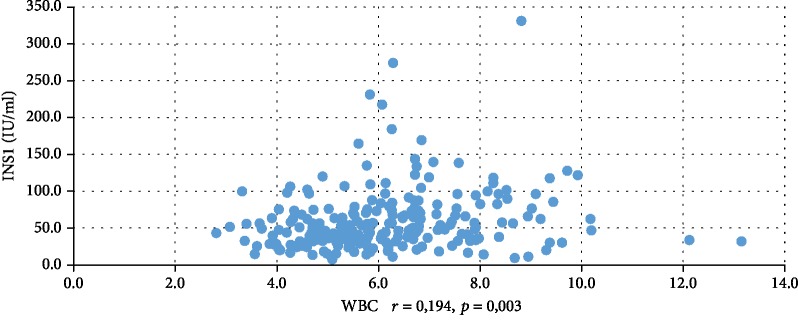
Correlation between INS1 and WBC.

**Figure 13:**
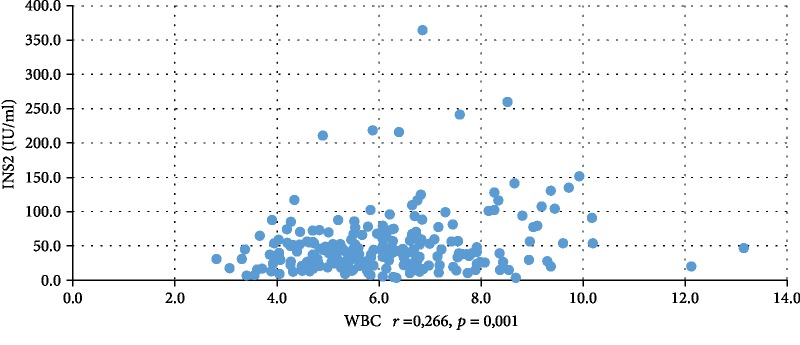
Correlation between INS2 and WBC.

In addition, WBC was positively correlated with serum insulin concentration at 0 min, 60 min, and 120 min during the oral glucose tolerance test (INS0: *r* = 0,221, *p* = 0,001; INS1: *r* = 0,194, *p* = 0,003; INS2: *r* = 0,022, *p* = 0,001), testosterone (*r* = 0,130, *p* = 0,046), androstenedione (*r* = 0,212, *p* = 0,001), and DHEAS (*r* = 0,178, *p* = 0,006) and negatively correlated with serum progesterone (*r* = −0,204, *p* = 0,002), estradiol (*r* = −0,140, *p* = 0,032), and SHBG (*r* = −0,308, *p* < 0,001). CRP was positively correlated only with serum insulin concentration at 0 min, 60 min, and 120 min during the oral glucose tolerance test (INS0: *r* = 0,343, *p* < 0,001; INS1: *r* = 0,276, *p* = 0,001; INS2: *r* = 0,320, *p* < 001) and negatively correlated with progesterone (*r* = −0,194, *p* = 0,030) and SHBG (-0,244, *p* = 0,005) concentrations (Tables 2 and 3). We also estimated positive correlation between BMI and serum CRP and WBC concentration. Significant correlations of the study parameters were also presented in Figures 1–13.

In order to determine the influence of several variables on the CRP and WBC results, stepwise logistic regression was performed using an alpha of *p* < 0, 05 for adding or removing predictors from the model. The variables explaining the analysis were testosterone, DHEAS, androstenedione, insulin, glucose, SHBG, and BMI. The final model included variables (Table 4 below), which explained 27% of CRP variation. The model fits the data well (*F* = 10,141, *p* < 0,001). CRP values are positively associated with BMI (beta = 0,374, *p* < 0,001) and insulin level (INS1) (beta = 0,282, *p* = 0,004). The variables included in the model explained only 16% of the WBC diversity. The proposed regression model proved to be well suited to the data (*F* = 10,671, *p* < 0,001) (Table 5) WBC results are negatively associated with SHGB (beta = −0,284, *p* < 0,001) and positively associated with testosterone (beta = 0,163, *p* = 0,024) and BMI (beta = 0,157, *p* = 0,047).

## 4. Discussion

These data suggest that chronic low-grade inflammation, in particular elevated WBC and CRP, occurs in PCOS. More specifically, women with PCOS have statistically significant higher WBC and CRP concentrations in comparison with their normal-ovulating, nonhyperandrogenic, age- and BMI-matched peers. These findings confirm what has been reported by a large number of different studies evaluating various populations of women with PCOS [17, 18].

The first data come from the study by Kelly et al. [11], who compared only 17 women with PCOS and 14 healthy controls. They found increased serum CRP in the study group, and it remained significant when age and BMI were accounted for. Elevated CRP levels were also found by Tola et al. [19], Souza dos Santos et al. [20], and Orio et al. [21]. In the meta-analysis of 31 clinical trials, conducted by Escobar-Morreale et al. [10], they concluded that CRP in women with PCOS is on average 96% (95% CI: 71-122%) higher than that in control groups. After adjustment of BMI, which has a significant impact on inflammatory process, an additional analysis was performed using data from 26 studies and CRP remained increased by 102% (95% CI: 73-131%).

Another marker of chronic inflammation is white blood cell count, and even modest elevations of WBC are associated with cardiovascular risk. In our study, WBC was statistically significantly higher in the PCOS group than in healthy controls. Similar results were obtained by Papalou et al. [22], Orio et al. [21], and Herlihy et al. [23]. In the study by Tola et al. [19], CRP was statistically significantly higher, but WBC was distributed homogenously between the two groups.

A large number of studies have shown the positive correlation between CRP, WBC, insulin resistance, BMI, and visceral fat [21, 22, 24]. Nevertheless, there is still uncertainty whether the inflammation is due to the PCOS itself or to the insulin resistance and obesity. Stepwise multiple regression analysis was carried out in the entire sample to assess the predictor factors of WBC and CRP. This analysis revealed BMI and INS1 as the main predicting factors of CRP and BMI, and testosterone and SHBG were predictors of WBC. In the first model, the variables explained 27% and in second model 16% of variation of aforementioned factors.

Our results are in agreement with the study by Phelan et al. [13]. According to his data, stepwise backward multiple regression revealed HOMA-IR, PCOS status, and BMI to be independent predictors of WBC, explaining 12,1%, 6,25%, and 2,5% of the total variance, respectively.

Despite that, we expected that such variables as insulin, testosterone, and BMI will explain WBC and CRP to a greater extent. We think that some factors could influence the results. Firstly, we calculated BMI and did not measure waist-to-hip ratio (WHR). Secondly, many factors could influence the model, e.g., smoking, hypertension, use of oral contraceptives, physical exercise, and alcohol consumption. Finally, the study population and ethnicity could play a role.

Although in our study the mean value of body mass index was normal (<25 kg/m^2^ in women with PCOS and in controls), the catalytic role of BMI was seen to be one of the main predicting factors of WBC and CRP when we applied multiple regression analysis in the whole sample. The other important predicting marker of CRP was insulin concentration.

More specifically, BMI and insulin resistance are indicated by many authors as the most important factors responsible for chronic low-grade inflammation status in PCOS women and consequently initiation and progression of atherosclerosis [21, 22, 25, 26].

In the study by Orio et al. and by Papalou et al., it was found that insulin resistance and obesity, not hyperandrogenemia, were probably the main factors responsible for the increase of leukocytes in patients with PCOS [21, 22], but contradictory results were found by Phelan et al. [13]. In contrast to the study presented by Orio and Papalou, our analysis demonstrates that hormonal levels are also predictors of leukocyte count in women with PCOS, suggesting that leukocyte increase and not CRP is affected by circulating androgens. In our data, WBC correlated positively with total testosterone, androstenedione, DHEAS, and in multiple regression analysis, testosterone was one of the main three predictor factors of leukocyte count. In our study, another predicting factor of WBC was SHBG, which is commonly associated with insulin resistance but also for diabetes type II and hypertension [27, 28].

Similar conclusions from this research were drawn also by Phelan et al. [13]. In his study, hsCRP and other cytokines correlated with insulin resistance, but increased WBC was observed in women with PCOS even when compared to equally obese and insulin-resistant women. It is possible that hyperandrogenemia individually or in combination with central adiposity and IR might explain leukocytosis. The exact mechanism has not been fully elucidated. So far, androgen receptors have been identified in lymphoid and nonlymphoid cells of the thymus and bone morrow [29] and in various human leukocytes with a particularly high expression in neutrophils [30]. There are also many research studies that indicate therapeutic activity for androgens against human leukemia cell lines in vitro and in vivo [31]. This could provide a possible explanation that androgen plays an important part in the development and activation of leukocytes and low-grade inflammation.

In summary, we suggest that PCOS is associated with increased WBC and CRP concentrations, which supports the evidence that PCOS is associated with low-grade inflammation. The main predicting factors of increased CRP are BMI and insulin resistance, but there is a relationship between WBC count in PCOS and androgen concentration itself so that inflammation may be mediated not only through adiposity but also through increased androgen concentration. However, due to many factors that can affect WBC and CRP levels, further studies are needed to understand the precise mechanism of chronic low-grade inflammation in women with PCOS.

## Figures and Tables

**Table 1:** Anthropometric, hormonal, and metabolic characteristics of PCOS patients and controls.

	PCOS (*N* = 200)		Controls (*N* = 105)		*p*
	Mean ± SD	Range	Mean ± SD	Range	
Age (years)	25, 84 ± 5, 42	18-40	24, 70 ± 6, 05	18-40	*p* = 0, 09
BMI (kg/m^2^)	24, 86 ± 4, 97	18-38	23, 44 ± 3, 57	18-36	*p* = 0,061
CRP (mg/dl)	2, 30 ± 3, 2	0,5-15,80	1, 31 ± 1, 48	0,22-7,50	*p* = 0,014^∗^
WBC (10 cells/ml)	6, 30 ± 1, 71	3, 81 ± 13, 15	5, 66 ± 1, 12	3,40-8,43	*p* = 0,019^∗^
FSH (mIU/ml)	4, 96 ± 1, 31	2,02-11,97	5, 49 ± 1, 67	2,8 0-12,00	*p* = 0,024^∗^
LH (mIU/ml)	7, 19 ± 3, 93	2,25-19,30	5, 02 ± 2, 09	1,44-11,04	*p* < 0,001^∗^
E2 (pg/ml)	41, 75 ± 23, 61	25,00-171	53, 14 ± 37, 11	19,00-218	*p* = 0,006^∗^
PRL (ng/ml)	30, 05 ± 13, 49	6,71-79,89	29, 13 ± 11, 26	8,13-63,26	*p* = 0,938
T (ng/ml)	0, 49 ± 0, 17	0,23-1,24	0, 36 ± 0, 11	0,13-0,54	*p* < 0,001^∗^
SHBG (nmol/l)	54, 84 ± 29, 87	9,20-190,80	68, 27 ± 33, 50	20,90-186,10	*p* < 0,001^∗^
A (ng/ml)	3, 89 ± 1, 50	1,11-10,00	2, 7 2 ± 0, 88	1,00-4,00	*p* < 0,001^∗^
DHEAS (*μ*mol/l)	9, 36 ± 3, 64	2,23-19,88	3, 33 ± 0, 70	1,25-4,46	*p* < 0,001^∗^
TSH (mIU/l)	1, 64 ± 0, 82	0,36-4,38	1, 62 ± 0, 83	0,39-4,40	*p* = 0,910
fT4 (pmol/l)	12, 77 ± 1, 50	9,14-17,87	13, 0 ± 2, 04	9,20-19,70	*p* = 0,087
17-OHP (ng/ml)	2, 02 ± 0, 81	0,20-5,99	1, 12 ± 0, 56	0,24-1,68	*p* ≤ 0,001^∗^
Progesterone (pg/ml)	4, 14 ± 5, 39	0,10-29,41	6, 81 ± 5, 37	3,19-20,18	*p* ≤ 0,001^∗^
GPO (mg/dl)	83, 20 ± 5, 76	61-101	84, 51 ± 6, 76	66,00-108,00	*p* = 0,120
GP1 (mg/dl)	142,65 ± 29, 30	66-219	125,58 ± 28, 41	57,00-178	*p* = 0,017^∗^
GP2 (mg/dl)	103,88 ± 27, 07	46-191	96, 74 ± 24, 80	51,0-167,0	*p* = 0, 03^∗^
INS0 (IU/ml)	7, 02 ± 3, 89	1,40-32,00	6, 0 2 ± 2, 69	1,2-16,20	*p* = 0,062
INS1 (IU/ml)	61, 64 ± 45, 12	5,80-330,8	45, 16 ± 21, 72	11,20-106,0	*p* = 0,004^∗^
INS2 (IU/ml)	53, 59 ± 46, 15	5,5-364,90	35, 99 ± 20, 88	5,10-117,20	*p* ≤ 0,001^∗^
Total cholesterol (mg/dl)	180,29 ± 33, 85	108-265	175,01 ± 29, 17	105-255	*p* = 0,151
Triglycerides (mg/dl)	93, 96 ± 47, 23	36-340	75, 50 ± 27, 59	32-201	*p* = 0,003^∗^
HDL cholesterol (mg/dl)	57, 22 ± 14, 81	25-103	73, 62 ± 24, 10	35-144	*p* ≤ 0,001^∗^
LDL cholesterol (mg/dl)	105,20 ± 27, 87	34-187	84, 59 ± 31, 90	37-170	*p* ≤ 0,001^∗^

^∗^ *p* < 0, 05: statistically significant.

**Table 2:** Correlation between WBC and BMI and hormonal and biochemical parameters.

Parameter	*r* = spearman correlation coefficient	*p* values
FSH (mIU/ml)	-0,062	0,344
LH (mIU/ml)	0,021	0,751
E2 (pg/ml)	-0,049	0,421
PRL(ng/ml)	-0,044	0,508
T (ng/ml)	0,130	0,046^∗^
SHBG (nmol/l)	-0,308	0,001^∗^
A (ng/ml)	0,212	0,001^∗^
DHEAS (*μ*mol/l)	0,178	0,006^∗^
TSH (mIU/l)	0,042	0,527
fT4 (pmol/l)	-0,006	0,922
17-OHP (ng/ml)	-0,007	0,918
Progesterone (pg/ml)	-0,204	0,002^∗^
GPO (mg/dl)	-0,062	0,345
GP1 (mg/dl)	0,054	0,411
GP2 (mg/dl)	0,096	0,146
INS0 (IU/ml)	0,221	0,001^∗^
INS1 (IU/ml)	0,194	0,003^∗^
INS2 (IU/ml)	0,220	0,001^∗^
BMI (kg/m^2^)	0,266	0,001^∗^

^∗^ *p* < 0, 05: statistically significant.

**Table 3:** Correlation between CRP and BMI and hormonal and biochemical parameters.

Parameter	*r* = spearman correlation coefficient	*p* values
FSH (mIU/ml)	-0,110	0,209
LH (mIU/ml)	-0,029	0,745
E2 (pg/ml)	0,044	0,620
PRL (ng/ml)	-0,143	0,109
T (ng/ml)	-0,034	0,696
SHBG (nmol/l)	-0,244	0,004^∗^
A (ng/ml)	-0,066	0,470
DHEAS (*μ*mol/l)	-0,093	0,287
TSH (mIU/l)	0,111	0,207
fT4 (pmol/l)	-0,153	0,081
17-OHP (ng/ml)	-0,094	0,322
Progesterone (pg/ml)	-0,194	0,030^∗^
GPO (mg/dl)	0,063	0,475
GP1 (mg/dl)	0,091	0,304
GP2 (mg/dl)	0,169	0,055
INS0 (IU/ml)	0,343	0,001^∗^
INS1 (IU/ml)	0,276	0,001^∗^
INS2 (IU/ml)	0,320	0,001^∗^
BMI (kg/m^2^)	0,339	0,001^∗^

^∗^ *p* < 0, 05: statistically significant.

**Table 4:** The predictors of CRP variation.

Predictors	*B*	Standard error	Beta	*t*	*p*
	-2,214	1,514		-1,462	≤0,001
BMI	0,214	0,055	0,374	3,893	≤0,001
INS1	0,017	0,006	0,282	2,962	≤0,001

**Table 5:** The predictors of WBC variation.

Predictors	*B*	Standard error	Beta	*t*	*p*
	5,053	0,858		5,889	≤0,001
SHGB	-0,016	0,004	-0,284	-3,581	≤0,001
Testosterone	1,662	0,730	0,163	2,277	0,024
BMI	0,055	0,028	0,157	1,998	0,047

## Data Availability

The data used to support the findings of this study are available from the corresponding author upon request.
